# Evaluating the Effects of Land Use Planning for Non-Point Source Pollution Based on a System Dynamics Approach in China

**DOI:** 10.1371/journal.pone.0135572

**Published:** 2015-08-12

**Authors:** Peng Kuai, Wei Li, Nianfeng Liu

**Affiliations:** 1 School of Environment, Beijing Normal University, Beijing, 100875, China; 2 School of Environment, Huazhong University of Science & Technology, Wuhan, 430074, China; Tennessee State University, UNITED STATES

## Abstract

Urbanization is proceeding rapidly in several developing countries such as China. This accelerating urbanization alters the existing land use types in a way that results in more Non-Point Source (NPS) pollution to local surface waters. Reasonable land use planning is necessary. This paper compares seven planning scenarios of a case study area, namely Wulijie, China, from the perspective of NPS pollution. A System Dynamics (SD) model was built for the comparison to adequately capture the planning complexity. These planning scenarios, which were developed by combining different land use intensities (LUIs) and construction speeds (CSs), were then simulated. The results show that compared to scenario S1 (business as usual) all other scenarios will introduce more NPS pollution (with an incremental rate of 22%-70%) to Wulijie. Scenario S6 was selected as the best because it induced relatively less NPS pollution while simultaneously maintaining a considerable development rate. Although LUIs represent a more critical factor compared to CSs, we conclude that both LUIs and CSs need to be taken into account to make the planning more environmentally friendly. Considering the power of SD in decision support, it is recommended that land use planning should take into consideration findings acquired from SD simulations.

## Introduction

Urbanization is proceeding rapidly in several developing countries such as China [1,2]. The Chinese urban population ratio grew from 36.2% to 53.4% between 2000 and 2013 [3]. It has been predicted that the urban population ratio in China will reach 60% by 2020 [4]. During this process, the existing land use is often irreversibly changed. For example, agricultural land is changed into urban land, which entails the removal of vegetation as well as the conversion of pervious areas into impervious surfaces [5].

Generally, developing countries will encounter the same challenges of inducing more Non-Point Source (NPS) pollution due to these land uses change [6]. In contrast to point source pollutants that enter waterways through pipes or channels, NPS pollutants are contaminants that are diffused in the environment and cannot be traced to a point location [6]. NPS pollution may exert very serious effects on water environments [7,8]; it is considered as one of the main causes of rivers failing to meet environmental requirements [9]. Many researchers have found that NPS runoff contains various kinds of pollutants, such as suspended solids, heavy metal pollutants, and hydrocarbon compounds [10,11], and also possesses very high pollution levels. For example, Hou et al. (2009), Yin et al. (2010), and Wang et al. (2013) found that the concentrations of suspended solids, chemical oxygen demand, biochemical oxygen demand, total nitrogen, and total phosphorous in the Chinese urban runoff exceeded class V of the Environmental Quality Standards for Surface Water (GB3838-2002) [12–14].

In an urbanization context, there are two ways that land use changes can influence NPS pollution. First, land use conversions alter the surface runoff hydrograph and thus increase storm water runoff volumes and peak flows [5,15]. Second, urbanization induces an increase in the local population and increases in socioeconomic activities, which in turn increase the generation of waste and pollutants in catchment areas [15]. These two mechanisms are further influenced by different planning choices regarding Land Use Intensity (LUI) and Construction Speed (CS). In the current study, LUI refers to different land use proportions which are critical to surface runoff hydrographs and are widely reported in the literature [16–19]. Although CS is critical for the increment of population size and socioeconomic activities, no previous studies have investigated its effects or the combination of effects from both LUI and CS. Furthermore, there is currently no consensus regarding the relative priority of LUI and CS.

Until now, many distributed and physically based watershed models, such as the water management model (SWMM) [20], the soil and water assessment tool (SWAT) [21,22], the hydrological simulation program–fortran (HSPF) [23], etc., have been used for assessing NPS pollution and show advantages in revealing the subtle physical mechanisms in terms of the rainfall-runoff process, soil erosion and the sediment transport process, pollutant migration and the transformation process, etc [24]. To assess these processes, a large number of continuous and synchronous data with respect to non-point source runoff and pollution are needed to make precise parameter estimations [25], however this type of data is quite scarce in China. Only by more experiments and field observations could these methods become more practicable [24], which is obviously costly in terms of time and money. System dynamics (SD) models, however, are not used for precise predictions but for revealing the defferences between planning scenarios in terms of their pollution effects through a systematic and long-term dynamic perspective [26,27], thus having potential to assess NPS pollution for where detailed runoff and pollution data are lacking.

When evaluating NPS pollution in a land use planning context, there are often extensive interactions among various planning elements [28], which make the system structures, behaviors, and the on-going development quite complex. As very complex emergent behavior can arise from a set of fairly simple underlying dynamics or rules [28], we can simulate complex system by integrating the simple causal/feedback loops based upon the interactions of the system elements. System dynamics (SD) provide a platform for such integration and simulation [29], and can accommodate the complexity, nonlinearity, and feedback loop structures that are inherent in social and physical systems [30]. An SD model aims at elucidating how system elements and decision making correlate with each other and affect the performances of a given system [31]. There are a number of studies that recount the detailed history of SD [30,32,33]. In an SD model, the system structure can be visually depicted by using professional software, e.g., Vensim PLE Plus [34], and causal/feedback loops, which are the most important elements in model simulations, can be observed [35]. There is an increasing number of studies on the application of SD models in urban development, land use management, and integrated sustainable management systems [29,35–37]. Here we use an SD model to assess land use planning in terms of the NPS pollution aspect to promote sustainable urban development.

This study aims to fill the gaps in the literature by evaluating the complex planning system and the combined effects of LUI and CSs on NPS pollution via an SD model. A case study is conducted for Wulijie, a small town in Wuhan City, China. The complex and inter-dependent relationships between/among the various components within the land use and NPS pollution subsystems are investigated. A simulation and comparison of the planning scenarios in terms of land use types, proportions and construction speeds are conducted. The findings are expected to assist in the decision-making process of local governments concerning the sustainable construction of small towns.

## Study Area and Data Sources

### 2.1 Site Description

The town of Wulijie is located in the middle-eastern part of Wuhan City, China. The total area of the town is 22.86 square kilometers (2286 ha). In 2010, the resident population was 19,000. Agricultural land, industrial land, and commercial service land account for 1716 ha, 37.4 ha, and 7.4 ha, respectively (Table 1).

**Table 1:** Land use in Wulijie in 2010.

Types	Area (ha)	Proportion (%)
**Agricultural land**	1716	75.1
**Industrial land**	37.4	1.6
**Commercial service land**	7.4	0.3
**Other land for construction** ^**a**^	305.1	13.3
**Other land for non-construction** ^**b**^	220.1	9.6
**Total**	2286	100.0

Note: ^a^Other land for construction includes urban and rural residential land, roads, railways, land for public facilities, *etc*.

^b^ Other land for non-construction includes water, woodland, grassland, *etc*.

Around and within Wulijie exists an abundance of water systems exist. Tangxun Lake is north of the town and represents the largest urban lake in China. Liangzi Lake, a scenario drinking water source for Wuhan, is located to the south of Wulijie. In addition, there are several reservoirs and rivers within the town. These water systems provide important ecological services to Wuhan City such as supplying water, maintaining biodiversity, sequestering carbon, and attracting tourism. However, any type of activity in a catchment that changes the existing land use has a direct impact on the quantity and quality characteristics of the water environment [5], and hence the land use planning of Wulijie is a considerable risk to the local water environment. Due to the abundant water systems and their important ecological services as well as the on-going land use planning, this area was a good candidate for this study.

### 2.2 Land use Planning

According to the draft plan (from 2010 to 2050) of Wulijie town, the main planning objectives include improving living conditions and attracting migration of population and industries from other parts of Wuhan City. The plan will entail the transformation of most of the former land use types, including changing agricultural land uses to industrial and commercial land uses in addition to constructing many new city communities with a large number of residential buildings.

Generally, top-down land use planning needs to include a determination of planning elements, such as LUI and CS, which are the main driving forces for the construction and induced NPS pollution. The planning scenarios were developed based on different combinations of LUIs and CSs (Table 2). Each of the LUIs and CSs was assigned a three-level gradient from low to high (for LUIs) or from slow to fast (for CSs). A lower LUI indicates a lower rate of agricultural land use converted to other land uses, such as industrial, residential, or commercial land use. Similarly, a slower CS indicates a slower increment of population size and socioeconomic activities. We assume that there are only slow changes in the CS aspect when the LUI is marked as “Low”; because in the context of China, it is quite unrealistic for a place which to maintain current land use types while simultaneously having a high construction speed; accordingly, the combination of Low (LUI) and Medium (CS), and Low (LUI) and Fast (CS), are not taken into account (Table 2).

**Table 2:** Planning scenarios according to different combination of LUIs and CSs.

Elements and levels		LUIs		
		Low^a^	Medium	High
**CSs**	**Slow** ^**b**^	S1 (BAU)	S2	S3
	**Medium** ^**b**^	/	S4	S5
	**Fast** ^**b**^	/	S6	S7

Note: ^a^The “Low” level of LUI is set as “business as usual” (BAU), where the land use maintains the levels of 2010.

^b^The planning interval is from 2010 to 2050; however the main task (i.e., finishing the transformation of existing land use) could be completed by the year 2050, 2040, or 2030, which are defined as a “Slow,” “Medium,” and “Fast” construction speed (CS), respectively.

Based on all of the other combinations, seven planning scenarios were developed; for example, scenario S1 is a Low (LUI) and Slow (CS) combination which represents a no planning scheme, also called business as usual (BAU); S2 is a Medium (LUI) and Slow (CS) combination for which 18.2% of existing agricultural land will be transformed into other land use types such as industrial land, commercial land, etc., and this transformation would be finished at the end of the planning (2050); S4 is a Medium (LUI) and Medium (CS) scenario for which the same proportion of existing agricultural land will be transformed and this transformation would be finished a little earlier in the planning process (2040). Specific settings for each scenario are shown in Table 3 and Table 4.

**Table 3:** Scenario settings for S1-S4.

Ratio of land use accounting for total area (TA) (%)	S1^a^		S2		S3^b^		S4	
	2010	2050	2010	2050	2010	2050	2010	2040–2050^c^
**Ratio of agricultural land (AL)**	75.1	75.1	75.1	56.9	75.1	38.7	75.1	**5**6.9
**Ratio of industrial land (IL)**	1.6	1.6	1.6	4.9	1.6	8.2	1.6	4.9
**Ratio of commercial land (CL)**	0.3	0.3	0.3	1.9	0.3	3.4	0.3	1.9
**Ratio of other lands for construction (OLC)**	13.3	13.3	6.1	23.0	13.3	40.0	13.3	23.0
**Ratio of other land for non-construction (ONLC)**	9.6	9.6	16.9	13.3	9.6	9.7	9.6	13.3

Note: ^a^The ratios in column S1 represent a BAU variation manner of the land resources. The agricultural lands that were converted were then assigned to industrial, commercial, and other land for construction according to their initial proportions (accounting for the total area), which were changed to 4.1%, 0.8%, and 34.1%, respectively. For other land for non-construction, we assumed that there was no area transformed from the agricultural land.

^b^The ratios of 38.7%, 8.2%, 3.4%, 40%, and 9.7% for AL, IL, CL, OLC, and ONLC, respectively, in the S3 columns are from the draft plan.

^c^ The time spans from 2040 to 2050 (or from 2030 to 2050) were set according to different construction speeds (CSs), and the corresponding ratios of each type of land use were kept constant during the period.

**Table 4:** Scenario settings for S5-S7.

Ratio of land use accounting for total area (TA) (%)	S5^a^		S6		S7^a^	
	2010	2040–2050^b^	2010	2030–2050^b^	2010	2030–2050^b^
**Ratio of agricultural land (AL)**	75.1	38.7	75.1	56.9	75.1	38.7
**Ratio of industrial land (IL)**	1.6	8.2	1.6	4.9	1.6	8.2
**Ratio of commercial land (CL)**	0.3	3.4	0.3	1.9	0.3	3.4
**Ratio of other land for construction (OLC)**	13.3	40.0	13.3	23.0	13.3	40.0
**Ratio of other land for non-construction (ONLC)**	9.6	9.7	9.6	13.3	9.6	9.7

Note: ^a^The ratios of 38.7%, 8.2%, 3.4%, 40%, and 9.7% for AL, IL, CL, OLC, and ONLC, respectively, in the S5, and S7 columns are from the draft plan.

^b^ The time spans from 2040 to 2050 (or from 2030 to 2050) were set according to different construction speeds (CSs), and the corresponding ratios of each type of land use were kept constant during the period.

A technical consultation meeting was arranged to discuss the specific settings for the draft plan, which represents the desires of the local governmental authorities. However, some environmental experts argued that this might be a radical option and would induce serious NPS pollution in the future. In this context, a compromise was made by averaging the current levels (in 2010) and the governmental options. The compromise values are 56.9%, 4.9%, 1.9%, 23.0%, and 13.3% for AL, IL, CL, OLC, and ONLC, respectively.

### 2.3 Data Sources

Generally, the data used in the SD model come from the following sources: a review of materials in yearbooks, governmental reports, and published research papers; site surveys/monitoring and interviews, and assumptions according to parameter relationships; and expert consultants and brainstorming [26]. It should be noted that an SD model does not pursue precise data because the model is not used for precise predictions about the future scenarios [26]. In this context, many data sources are combined in an SD model (See Table 5 [13,38–43] and details in the Supporting Information).

**Table 5:** Data sources used for assignment and validation of the model.

Parameter sort	Data sources	Notes
Land use ratios that account for the total area under different scenarios	Experiential functions, expert consultants	The logistic curves are assigned according to the law of diminishing returns and scarce resource restrictions (Sun, 2012)
Runoff coefficient of a given land use type	Referencing the related design manual and regulations	The Chinese Academy for Environmental Planning, 2004; Beijing municipal engineering design & research institute, 2004
Pollution intensities of different land use types	Literature review	Yin, 2010; Liang and Qin, 2013; Tang, et al., 2010; Li and Li, 2013
Decrement of NPS pollution (DNPSP)	Experiential functions, expert consultants, literature review	The Chinese Academy for Environmental Planning, 2004
Statistical values for historical fit test	Site surveys of the Wulijie area	

Note: specific settings of the model are shown in Table A in S5 Text.

## The SD-Based Method

### 3.1 System Definition

**Fig 1:**
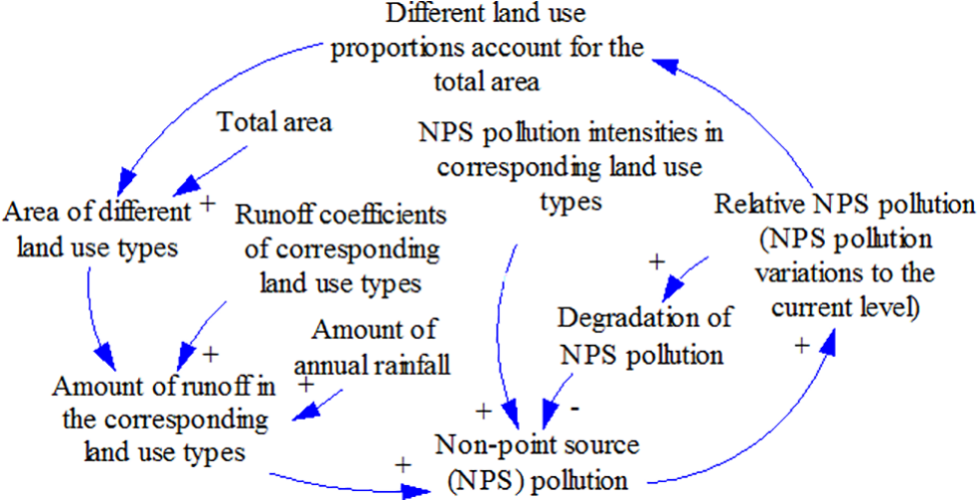
Conceptual model of the land use-NPS pollution system. Note: “+” and “–” represent positive and negative feedbacks.

The current study models NPS pollution variations for different land use planning scenarios. Different land use types and proportions as well as their corresponding runoff and NPS pollution intensities were selected as system parameters according to the conceptual model (as shown in Fig 1). Among these parameters, the land use proportions were selected as critical policy variables that drive the NPS pollution variations.

There are two main causal loops in Fig 1; the first is between the “Area of different land use types” and “NPS” pollution. In this loop, the causal relationship depends on the specific proportions of different land use types. The basic assumption in this model is that greater proportions of land for construction induce more runoff (due to more impervious underlying surfaces) and enhanced NPS pollution intensities (due to greater socioeconomic activity) [5]. In turn, higher levels of predicted NPS pollution should lead decision makers to choose planning scenarios with fewer negative impacts. The other causal loop is between “NPS” pollution and “degradation of NPS” pollution, which shows a negative relationship within certain realms, i.e., more “NPS” pollution will induce more degradation (of “NPS” pollution) [44–46].

### 3.2 Developing the SD Model

**Fig 2:**
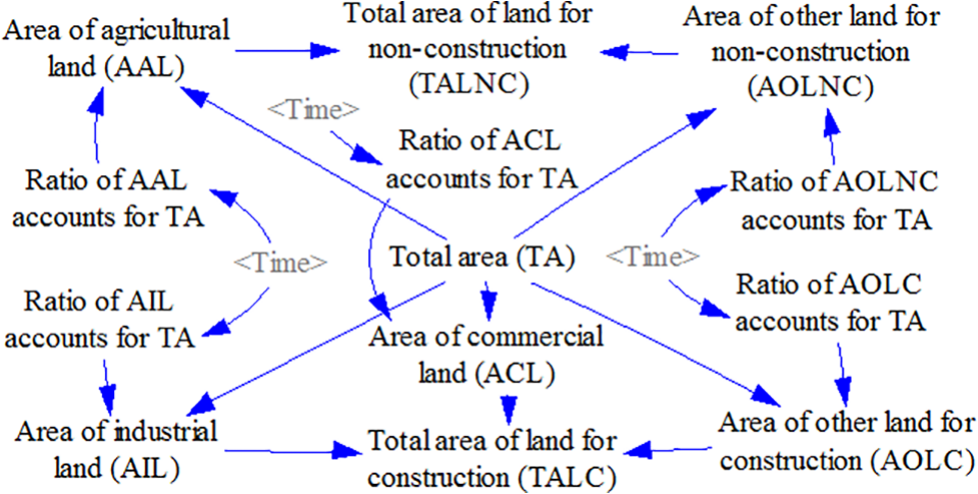
Flow diagram of the land use subsystem.

**Fig 3:**
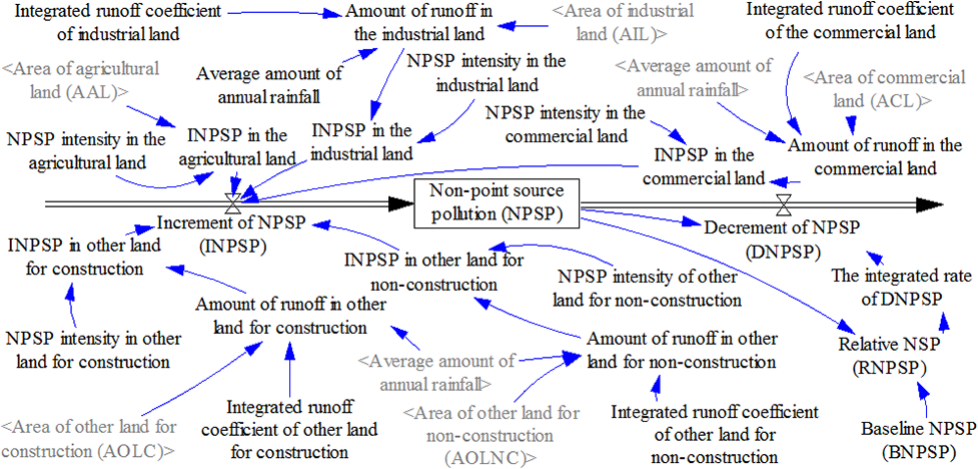
Flow diagram of the NPS pollution subsystem.

Based on the above conceptual model depicted in Fig 1, the land use subsystem and NPS pollution subsystem were developed using the Vensim PLE Plus software [34]. The time-step was 1 year and the time-span was from 2010 to 2050 (Figs 2 and 3).

#### 3.2.1 Land use Subsystem

In the case-study area, agricultural lands and other lands for non-construction are expected to decrease in area, whereas the land for construction is expected to grow, particularly industrial land, commercial land, and urban residential land (which is included in the “other land for construction (OLC)” category) (Fig 2). The future area for each type of land use is determined by a ratio accounting for the total area (TA), where TA is a constant whose value is 2286 ha. The ratios were set in the model as policy variables and will change over time according to different planning scenarios, where the initial and final values of the ratios should be determined during the planning process (Table 3 and Table 4). However, the planning stage does not make specific settings regarding how the ratios vary between the initial and final values. Considering that land resource utilization has always been subject to the law of diminishing returns and would be restricted when resources become scarce [38,47], the logistic function was employed to express the variation in land use ratios (see the specific settings in S1 Text).

#### 3.2.2 NPS Pollution Subsystem

NPS pollution was determined by the amount of runoff (AR), the pollution intensity [5], and the decrement factors. Here, we assume that all runoff and pollutants they carried will be transferred into local water bodies; by multiplying AR and the pollution intensities we obtain a rough estimation of the amounts of NPS pollutants that enter the water bodies. Then, by subtracting the degradation amounts, we obtain the accumulation amounts of NPS pollutants. (Fig 3).

To depict AR, an empirical equation is often used (see Formula 1) [13], where AR is determined by the amount of rainfall, land area, and the corresponding runoff coefficient, which varies for different land use types.

$$Q=\sum_{\text{j}=1}^{n}P*A_{\text{j}}*\rho_{\text{j}}*10^{-3},$$ (Formula 1)

In the above formula, Q is the amount of runoff (m^3^), *P* is the amount of annual rainfall (mm), *A*_*j*_ is the area of land use type j (m^2^), and *ρ*_j_ is the runoff coefficient corresponding to a given land use type j. It should be noted that climate change is a critical factor to the rainfall-runoff process, especially when the planning lasts in a long time horizon. However, climate change is of high uncertainty and is difficult to predict [48]. As in the current study, we mainly care about different land use types, and climate change is deemed as an external variable that will exert the same influences to all land use change scenarios. In this context, “P” is set as a constant, which is assigned by averaging the local annual historical rainfall data. Additionally, impervious surface is another critical factor to the runoff process. For example, Wu et al., (2013) found that the quick-response surface runoff would be significantly increased (with an annual average increase of 10.5%) due to the increase in impervious areas [49]. Burn and Band (2000) found that the runoff ratio would change dramatically when impervious cover percent increases beyond 20% [50]. In the current study, runoff coefficients are employed to denote the runoff capacities of different land surfaces; the larger a runoff coefficient, the more impervious a surface is (see S2 Text).

With regards to pollution intensity, there are significant differences between different land use types [13,51,52]. However, it is difficult to obtain exact values of the intensity for a specific location. The data are either collected via a large number of outdoor experiments and monitors or by reviewing the literature for historical information [53]. In China, both sources of data are sparse [24], especially for small towns. In the current study, related literature was used to estimate values [13,41–43].

To make the simulation more convenient, a new index of pollution equivalence (PE) was introduced to integrate different pollutants. In the Chinese context, COD and NH_3_-N are two mandatory indices for water pollution control [54], and are employed for integration. The formula for PE is as follows:

$$PE=C_{C}/SC_{C}+C_{N}/SC_{N}$$ (Formula 2)

where C_C_ and C_N_ represent the pollution concentration (intensity) of COD and NH_3_-N of a given land use type, respectively. SC_C_ and SC_N_ represent the class II values of the Environmental Quality Standards for Surface Water (GB3838-2002) [55] (see the specific settings of PE in Table A in S3 Text).

With regard to the decrement of NPS pollution (DNPSP), more NPS pollution will induce a larger integrated rate of decrement in NPS pollution (IRDNPSP) [44–46]. Herein, IRDNPSP (1/year) represented the annual reduction rate in the pollution equivalence, which is a cumulative effect of daily degradation of pollutants for a given year, where the daily degradation is represented with degradation coefficients (1/day) for the pollutants under different water quality statuses [39]. A dynamic model, through which the cumulative effect of daily degradation of the pollutants was simulated, was developed to estimate IRDNPSP (see details in S4 Text).

### 3.3 Model Validation

In the current study, a historical fit test and a sensitivity test were conducted for the validation. The historical fit test was conducted to determine whether the simulated behavior of the model followed the historical behavior [29]. The mean absolute relative error (MARE) index was employed for the test [56], and the threshold for acceptable MARE values is 10% [57]. The sensitivity test was conducted to ensure that the model was robust at given variations of the parameters [56,58]. To conduct the test, the “one-at-a-time” method was used; this varies the value of one parameter while keeping the values of the other parameters constant [56]. All of the parameters with high uncertainties must be accounted for as well as the most excessive variations of their values [26]. The index of total sensitivity, “S”, was adopted for sensitivity tests [56], and the value of S should be lower than 1 to be considered acceptable [58].

## Results and Discussion

### 4.1 Model Validation Results

#### 4.1.1 Historical Fit Test

Changes in land use types constitute the most critical driving force for the variation in NPS pollution [5]. Therefore, in the current study, the areas of different land use types during 2010 and 2013 were selected for the historical fit test. The mean absolute relative errors (MAREs) for each of the variables were then calculated and are shown in Table 6. Because all of the MAREs were lower than 10%, we concluded that the model exhibited a good historical fit.

**Table 6:** Results of the historical fit test.

Time	AAL^a^		ACL^a^		AIL^a^		AOLC^a^		AONLC^a^	
	STV^b^	SIV^b^	STV	SIV	STV	SIV	STV	SIV	STV	SIV
**2010**	1717.01	1717.01	7.18	7.18	36.81	36.81	306.18	306.18	219.46	219.46
**2011**	1707.80	1696.80	8.38	7.46	44.81	38.25	310.17	318.15	215.48	219.46
**2012**	1667.20	1677.56	8.49	7.75	43.50	39.71	344.89	330.27	222.56	219.46
**2013**	1632.70	1659.23	9.97	8.04	47.15	41.18	376.20	342.51	220.62	219.46
**MARE**	0.7%		9.8%		9.0%		3.9%		0.9%	

Note: ^a^AAL, ACL, AIL, AOLC, and AONCL represent area of agricultural land, area of commercial land, area of industrial land, area of other land for construction, and area of other land for non-construction, respectively. The unit is ha.

^b^STV and SIV represent statistical values and simulated values of the variables, respectively. The STVs come from site surveys of the Wulijie area, and the SIVs come from the SD model built in the current study.

#### 4.1.2 Sensitivity Test

NPS pollution was selected as the stated variable to be validated. Three types of parameters, including different land use combinations [59], NPS pollution (NPSP) intensities, and integrated runoff coefficients (which are likely to have high uncertainties), were selected for the sensitivity test. The land use combinations, named I, II, III, IV, and V, were used in the test and were set as follows: for combination I, we assumed that all of the agricultural land (in 2010) was transformed to commercial land (CL), industrial land (IL), other land for construction (OLC), and other land for non-construction (ONLC) during 2010 and 2050 according to the initial land use proportions; for II, III, IV, and V, we assumed that all of the agricultural land (in 2010) was transformed to CL, IL, OLC, and ONLC, respectively. With respect to NPSP intensities and integrated runoff coefficients, both were adjusted by an increment of 100% or a decrement of 50% (see specific adjustments and the corresponding test results in Table 7). Because nearly all of the S-values were lower than 1, we concluded that the model is robust when there are variations in the parameter values.

**Table 7:** Results of the sensitivity test.

Parameters and adjustments^a^	S-value (%)				
	2010	2020	2030	2040	2050
**Combinations I of the land use types**	0.00	0.79	0.17	0.06	0.16
**Combinations II of the land use types**	0.00	0.67	0.27	0.92	0.89
**Combinations III of the land use types**	0.00	0.57	0.09	0.47	0.52
**Combinations IV of the land use types**	0.00	0.35	0.28	0.54	0.6
**Combinations V of the land use types**	0.00	1.11	0.83	0.74	0.73
**NPSP intensities in the different land use types (+100%)** ^**b**^	0.00	0.89	0.91	0.93	0.94
**NPSP intensities in the different land use types (-50%)** ^**b**^	0.00	0.80	0.82	0.84	0.85
**Integrated runoff coefficients of different land use types (+100%)** ^**b**^	0.00	0.48	0.56	0.61	0.65
**Integrated runoff coefficients of different land use types (-50%)** ^**b**^	0.00	0.43	0.52	0.59	0.62

Note: ^a^the above adjustments are conducted based on the BAU pattern.

^b^+100% and -50% represent an increment by 100% and a decrement by 50%.

### 4.2 Simulation of relative NPS Pollution (RNPSP)

In the current study, the focus was placed on variations in NPS pollution under different LUIs and CSs. The index of RNPSP, which is used to indicate the variation, is defined as follows:

$$RNPSP=NPSP_{i}/BNPSP$$ (Formula 3)

where NPSP_i_ represents the simulated NPS pollution at year I and BNPSP represents the baseline NPS pollution at year 2010.

Different scenarios, with respect to LUIs and CSs, were simulated and compared. Two criteria were used for the comparisons: one reflecting the environmental protection objective and one reflecting the development objective. The environmental criteria require that increases in NPS pollution would not exceed 50% during the planning process when compared with BNPSP; in other words, RNPSP should not exceed 1.5. The 50% value was selected because reports indicate that if pollution levels in the lakes of Wulijie increase by 50%, the surface water quality status would be degraded from Class III to Class IV of the Environmental Quality Standards for Surface Water (GB3838-2002), which renders the water in Liangzi Lake non-potable [60]. The developmental criteria reflect the development objective which prioritizes faster construction for faster socioeconomic development. A two-step process of assessing scenarios was conducted according to the specified criteria. First, the RNPSPs of different scenarios were simulated and compared; in this step, the scenarios that failed to meet the environmental protection objective were eliminated. Second, CSs were inspected for the scenarios retained in the first step, and the scenario with the fastest CS was then chosen as the “best” option.

**Fig 4:**
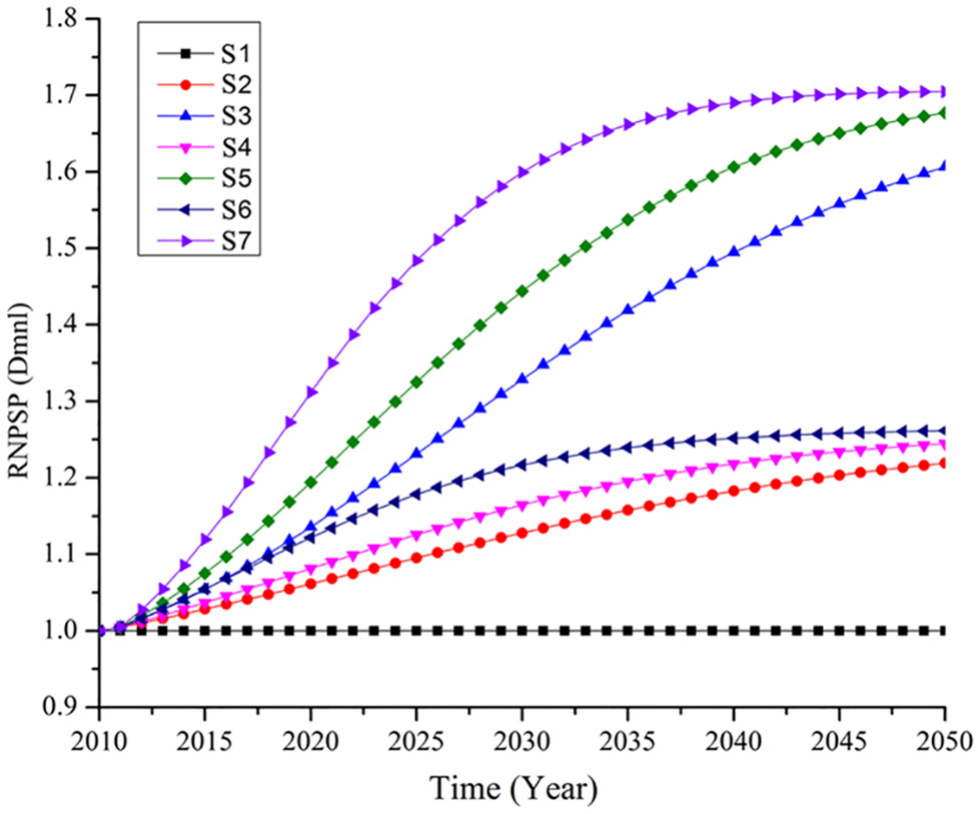
Simulation results of RNPSP under different scenarios. Note: S_i_ represents different scenarios (see Table 3 and Table 4); "Dmnl" is a default setting of the Vensim software package for SD models; it is used when a variable or parameter is dimensionless.

The simulation results are shown in Fig 4. This Fig shows that compared with scenario S1 (business as usual), all of the other planning scenarios will introduce more NPS pollution in Wulijie. The scenarios in decreasing order of pollution consequences are S7, S5, S3, S6, S4, and S2, and the corresponding RNPSP values in 2050 were simulated as 1.70, 1.68, 1.61, 1.26, 1.24, and 1.22, respectively. This indicates an increase in NPS pollution ranging from 22% to 70% compared with the initial NPS pollution status (in 2010).

The scenarios were sorted into two groups. One group included scenarios where NPS pollution increased by more than 50%, such as scenarios S7, S5, and S3, which increased RNPSP by 70%, 68%, and 61%, respectively. These scenarios will incur large risks to the local waters, especially to Liangzi Lake, which is the alternate drinking water source for Wuhan. Thus, these scenarios were eliminated in the first step of comparison. The other group includes scenarios where NPS pollution increased by less than 50%, such as S6, S4, and S2; these three scenarios were retained during the first step of comparison for meeting the environmental protection objective. CSs were then inspected for the remaining scenarios, and S6 was selected as the best scenario as it possesses the fastest CSs (Table 2). S6 was then proposed to the decision makers as a way to optimize the land use planning.

The simulation also found that LUI is a more critical factor than CS with respect to increased NPS pollution, which agrees with previous studies that highlight LUI [16–19]. Fig 4 shows that scenarios S7, S5, and S3 induce more NPS pollution than scenarios S6, S4, and S2; obviously, the scenarios with High LUI rather than with Fast CS introduce more NPS pollution to Wulijie. Thus, it is recommended that more attention be paid to LUI during land use planning. As A substantial amount of rural land will be inevitably transformed into urban land during the rapidly proceeding urbanization in the developing world [1,2]. Maintaining pervious cover is important to decrease storm water runoff volumes and peak flows and thereby further decrease NPS pollution [19]. It should be noted that although CS is not as important as LUI with respect to increased NPS pollution, it still needs serious consideration. The comparison between scenarios S2, S4, and S6 shows that faster CSs will obviously introduce more NPS pollution (Fig 4).

The current study shows the advantages of an SD model in comparing planning scenarios in a complex land use planning context. Models are used to simplify a complex system and can help develop an understanding of specific problems [26]. Although there are many types of models used for predicting NPS pollution such as the storm water management model (SWMM) [20], the soil and water assessment tool (SWAT) [21], and Geographic Information System (GIS) based models [18], SD models are more transparent and participatory as well as less costly in terms of time and money. For instance, the land use subsystem and NPS pollution subsystem and their complex interactions can be visually provided to decision makers and stakeholders through the use of the system flowchart. Additionally, future changes in system structures can be more easily addressed as there is a better initial understanding of the system structure [61]. SD models provide an additional advantage with their focus on making comparisons between different scenarios rather than making precise predictions [26,27]. This focus on comparisons means that simulations can be run without a large number of experiments and field observations required to make precise parameter estimations [25]. Finally, SD models facilitate communication between the modelers, decision makers, and stakeholders [26]. These features are critical in developing countries where data reserves, money, and time are lacking. Thus, it is recommended that land use planning decisions take into account findings acquired from SD simulations.

Nevertheless, there are some limitations in this study. For example, the pollution intensities of a given land use type are closely related to population size and socioeconomic activities, however, this relationship was not directly simulated in the current study; instead, previous studies concerning pollution intensities were reviewed [13,38–43]. In the future, a more comprehensive model will be required to explore the relationships between NPS pollution variation and land use change, population, and socioeconomic level. Additionally, climate change is critical to the rainfall-runoff process; however, it was not considered in the model due to its inherent uncertainties. To determine the uncertainties, more climate change scenarios are required [48]; however, this is quite difficult to obtain for small town areas because both global climate change trends as well as the town’s own specific features need to be considered. Instead, climate change is set as an external variable of the model and is assumed to exert the same influences to all land use scenarios. This assumption is obviously convenient, but further study should be conducted based on the IPCC’s research and more detailed field surveys.

## Conclusions

Urbanization is proceeding rapidly in several developing countries such as China. During this process, the existing land use is often irreversibly changed, which may pose significant risks to the local surface water by introducing additional NPS pollution. In this context, a reasonable land use plan is needed. The two factors of LUI and CS are critical in planning for increases of NPS pollution. However, only LUI has been highlighted in previous studies whereas CS is often totally neglected. Additionally, land use change due to urbanization represents a complex system with dynamic variations and extensive interactions; however, the methods used in previous studies such as SWMM, SWAT, and GIS are not designed to address these problems.

This study aims to fill the gaps in the literature by evaluating the complex planning system and combined effects of LUI and CS on NPS pollution via a SD model. A case study was conducted in Wulijie, a small town in Wuhan City, China. The complex and interdependent relationships between/among the various components within the land use and NPS pollution subsystems were revealed. Seven scenarios, which were developed by combining LUI and CS, were simulated and compared. The simulation of scenarios S2-S7 showed that compared with scenario S1 (business as usual), NPS pollution will increase by 22% to 70% by the end of the planning period (2050). Through comprehensive comparisons, scenario S6 (the combination of a medium LUI and a fast CS) was selected as the best option because it may induce relatively less NPS pollution while simultaneously maintaining a considerable development rate. Furthermore, although the simulation showed that LUI constitutes a more critical factor than the CS, both LUI and CS need to be taken into consideration in the decision making process.

In the present analysis, the SD method was used to simulate the effects of land use planning on NPS pollution. The SD method exhibited the advantages in addressing the planning complexity. Compared to alternative methods, the SD approach was more transparent and participatory as well as less time consuming and costly. Thus, it is recommended that land use planning decisions take into account findings acquired from SD simulations. Nevertheless, a more comprehensive model will be needed in the future to explore the relationships between NPS pollution variation and land use change, population, and socioeconomic level.

## Supporting Information

S1 Text Assigning the land use ratios that account for the total area under different scenarios. (DOC)

S2 Text Determining the runoff coefficients for different land use types. (DOC)

S3 Text Determining the pollution equivalence (PE) of different land use types. (DOC)

S4 Text Estimating the integrated rate of decrement in NPS pollution (IRDNPSP). (DOC)

S5 Text Assigning the model. (DOC)
