# Supplementary material for: Evaluating the Effects of Land Use Planning for Non-Point Source Pollution Based on a System Dynamics Approach in China
Source: PLoS One. 2015 Aug 12;10(8):e0135572. doi: 10.1371/journal.pone.0135572 (PMC4534394; doi:10.1371/journal.pone.0135572)
Supplement: S1 Text — (DOC) [file pone.0135572.s001.doc]

# S1 Text. Assigning land use ratios under different scenarios.

Table A shows the assignments for the ratios of different land use types under different scenarios. Based on the law of diminishing returns and scarce resource restrictions , we assumed that the variations of each land use type would exhibit an accelerating trend at first and deceleration afterwards. The logistic curve is classically used for depicting this variation trend in resource use contexts , and we employed it here for the assignment (see Eq. 1),

$Y=L/(1+\alpha*\text{EXP(}-\beta*(t-2010)))$ (Eq. 1),

Where Y represents the ratios of different land use types accounting for the total area, L is the final ratio, and t is the time variable. $\alpha$ and $\beta$ are the parameters to be determined. The values of the initial ratios (at 2010) and the final ratios (at the end of construction, *i.e.*, 2030, 2040, or 2050) were based on our settings for the different scenarios (see Table 3 and Table 4 in the manuscript).

**Table A. Assigning the land use ratios (%) (In part)**

| **Scenario** | **Indices** | **Assignments** |
| --- | --- | --- |
| **S1** | Ratio of AL accounting for TA | $51.3/(1+1.546*\text{EXP(}-0.026*(t-2010)))$ |
|  | Ratio of IL accounting for TA | $4.1/(1-0.317*\text{EXP(}-0.064*(t-2010)))$ |
|  | Ratio of CL accounting for TA | $0.8/(1+1.546*\text{EXP(}-0.064*(t-2010)))$ |
|  | Ratio of OCL accounting for TA | $34.1/(1+1.546*\text{EXP(}-0.064*(t-2010)))$ |
|  | Ratio of ONCL accounting for TA | 9.6 |

Note: for the other scenarios such S2, S3…S7, detailed assignment are presented in Table A in S5 Text.

# References

1. Sun Z (2012) Analysis of synergy effect of institution-technology-resource on ecology economy. Ecology and Environmental Sciences 21: 590-594.

2. Chen M (2003) Extension and Application of Logistic Curve. Operations Research and Management Science 12: 85-88.
