# Supplementary material for: Evaluating the Effects of Land Use Planning for Non-Point Source Pollution Based on a System Dynamics Approach in China
Source: PLoS One. 2015 Aug 12;10(8):e0135572. doi: 10.1371/journal.pone.0135572 (PMC4534394; doi:10.1371/journal.pone.0135572)
Supplement: S2 Text — (DOC) [file pone.0135572.s002.doc]

# S2 Text. Determining the runoff coefficients for different land use types.

The runoff coefficient of a given land use type was determined by referencing the related design manual and regulations (Table A).

**Table A**. Runoff coefficients for different land use types

| **Land use types** | **Runoff coefficients** |
| --- | --- |
| **Industrial land** | 0.7 |
| **Administrative and commercial service land** | 0.8 |
| **Other land for construction** | 0.60 |
| **Other land for non-construction** | 0.15 |

# References

1. The Chinese Academy for Environmental Planning (2004) The technical key points of surface water's environmental capacity.

2. Beijing municipal engineering design & research institute (2004) Design manual for water supply and drainage; 5, editor. Beijing: China Architecture & Building Press.
