# Supplementary material for: Evaluating the Effects of Land Use Planning for Non-Point Source Pollution Based on a System Dynamics Approach in China
Source: PLoS One. 2015 Aug 12;10(8):e0135572. doi: 10.1371/journal.pone.0135572 (PMC4534394; doi:10.1371/journal.pone.0135572)
Supplement: S3 Text — (DOC) [file pone.0135572.s003.doc]

# S3 Text. Determining the pollution equivalence (PE) of different land use types

Based on Formula 2 in the manuscript, the PE values of different land use types were calculated. It is difficult to obtain exact values of the pollution intensities of a given location due to the large amount of money, time, manual labor, and material resources required. As this was beyond the scope of this paper, we referenced the related literature for the assignment (Table A).

**Table A**. Pollution equivalence of different land use types

| **Land use types** | **Pollution intensities (mg/L)** | | **Pollution equivalence (Dimensionless)** | **References** |
| --- | --- | --- | --- | --- |
|  | COD | NH_3_-N |  |  |
| **Agricultural land** | 17.2 | 12.9 | 26.95 |  |
| **Industrial land** | 64.1 | 3 | 10.27 |  |
| **Commercial land** | 340.7 | 3.2 | 29.11 |  |
| **Other land for construction** | 121.1-645.4 | 3-6.6 | 20.07-69.43 |  |
| **Other land for non-construction** | 45.6 | 0.41 | 3.86 |  |
| **Class II of the Standards** | 15 | 0.5 | / |  |

# References

1. Liang L, Qin M (2013) Research on agricultural non-point souce pollution in China. Beijing: China Social Sciences Press.

2. Tang H, Huang S, Wu J, Qiu J (2010) Study on the removel effects of agricultural runoff pollutants in riparian buffer on condition of continuous rainfall. Journal of Shanghai Jiaotong University (Agricultural Science): 426-431.

3. Li H, Li J (2013) Research and application of quantitative methods on non-point source pollution in the watershed area. Beijing: Science press.

4. Yin C (2010) Theories and technologies of controlling urban non-point source pollution Beijing: China Architecture & Building Press.

5. Ministry of Environmental Protection of the Peoples' Republic of China (2002) Environmental quality standards for surface water (GB 3838-2002).
