# Supplementary material for: Evaluating the Effects of Land Use Planning for Non-Point Source Pollution Based on a System Dynamics Approach in China
Source: PLoS One. 2015 Aug 12;10(8):e0135572. doi: 10.1371/journal.pone.0135572 (PMC4534394; doi:10.1371/journal.pone.0135572)
Supplement: S4 Text — (DOC) [file pone.0135572.s004.doc]

# S4 Text. Estimate of the integrated rate of decrement of NPSP (IRDNPSP)

**(1) Defining the correspondences between water status and the relative NPS pollution (RNPSP)**

According to our survey, the baseline water statuses of Tangxun Lake and Liangzi Lake in 2010 were approximately Class III according to the Environmental Quality Standards for Surface Water (GB3838-2002). Correspondingly, we defined the RNPSP as equal to 1. Then, according to the water quality standards, we calculated the PEs (see Formula 2 in the manuscript) and the corresponding ratios of the PEs accounting for the Class III standard. Based on the ratios, we defined the correspondences between water status and the RNPSPs (see Table A).

**Table A. Defining the correspondence between water status and RNPSP**

|  | **Class II** | **Class III** | **Class IV** | **Class V** |
| --- | --- | --- | --- | --- |
| **COD (mg/L)** | 15 | 20 | 30 | 40 |
| **NH_3_-N (mg/L)** | 0.5 | 1.0 | 1.5 | 2.0 |
| **PE** | 2.00 | 3.33 | 5.00 | 6.67 |
| **Ratio of PE for Class III** | 0.6 | 1 | 1.5 | 2 |
| **RNPSP** | 0.6 | 1 | 1.5 | 2 |

**(2) Selecting every day’s degradation coefficients according to RNPSP**

Based on research by the Chinese Academy for Environment Planning, the corresponding degradation coefficients of COD and NH_3_-N were set as follows :

**Table B**. Degradation coefficients for the lakes

| **Water status** | **Degradation coefficients (1/d)** | |
| --- | --- | --- |
|  | COD | NH_3_-N |
| **Good** | 0.01-0.03 | 0.01-0.03 |
| **Medium** | 0.03-0.06 | 0.03-0.06 |
| **Poor** | 0.06-0.10 | 0.06-0.10 |

Note: the water statuses of Good, Medium, and Poor correspond to the water standards of Class II-Class III, Class III-Class IV, and Class IV-Class V, respectively.

The degradation coefficients of the pollutants for different RNPSPs were set as follows:

**Table C. RNP**SP and the corresponding degradation coefficients

|  | **RNPSP values and the corresponding degradation coefficients** | | | |
| --- | --- | --- | --- | --- |
| **RNPSP** | 0.6 | 1 | 1.5 | 2 |
| **Degradation coefficients (1/d)** | 0.01 | 0.03 | 0.06 | 0.1 |

Note: for the other RNPSPs, which are smaller than 0.6 or larger than 2, the corresponding degradation coefficients were set as 0.01 (1/d) and 0.1 (1/d), respectively. This setting was based upon research that shows that the increment in degradation coefficients is accompanied by degradation in water quality. However, this only occurs to a certain extent when the water statuses are maintained between Class II and Class V. Once this threshold is exceeded, the degradation coefficient remains almost constant .

**(3) Simulating the accumulative effect of daily degradation of the pollutants and calculating the integrated rate of decrement of NPSP (IRDNPSP)**

Based on the degradation coefficients (1/d) in Table C, a dynamic model was developed by using the professional software Vensim PLE Plus . This model was used to calculate the corresponding IRDNPSP (1/year), see Figure A and Table D.

**Figure A. Accumulative effect of daily degradation of the pollutants**

In the above model, “NPS pollution level” is set as a stock which is accumulated by the difference between “Increment of NPS pollution” and “Decrement of NPS pollution.” The initial value of “NPS pollution level” was set as “0,” i.e., no pollution at the beginning. It should be noted that the initial value of “NPS pollution level” would cause differences in the final results of “NPS pollution level” and “IRDNPSP.” For example, when the initial value was set as 0, 100, 200, or 300, the final values of “IRDNPSP” (when “Degradation coefficient” was set as 0.01, see Table D) were 0.733, 0.785, 0.818, and 0.842, respectively. Compared with the differences caused by the “Degradation coefficients” (see Line 80~81), the differences caused by the initial value of “NPS pollution level” were much smaller and can therefore be neglected.

We assumed that there was a constant increment of pollution (1 unit per day), thus the value “1” was assigned to “Increment of NPS pollution.” We also assumed that “Decrement of NPS pollution” was subject to “NPS pollution level.” The daily “Degradation coefficient” was set at 0.01 day^-1^, 0.03 day^-1^, 0.06 day^-1^, or 0.1 day^-1^ (Table D). The time interval of the model is from 1 to 365 with a time-step of 1 day, thus the difference of every day can be accumulated through a whole year.

**Table D. Assignment of the accumulative effect model**

| **Parameters** | **Functions** | **Units** |
| --- | --- | --- |
| **Decrement of NPS pollution** | “NPS pollution level” * “Degradation coefficients” | Dmnl |
| **Degradation coefficient** | 0.01/0.03/0.06/0.1 | 1/day |
| **Increment of NPS pollution** | 1 | Dmnl |
| **NPS pollution level** | INTEG (“Increment of NPS pollution” – “Decrement of NPS pollution”, 0) | Dmnl |

The simulation showed that when “Degradation coefficient” was 0.01, “NPS pollution level” at the 365th day was calculated as 97.422. Thus, the IRDNPSP was calculated as: “1 unit per day*365 days-97.422 units” / “1 unit per day*365 days”=0.733; similarly, IRDNPSPs under the “Degradation coefficients” of 0.03, 0.06, and 0.1 were calculated as 0.9087, 0.9543, and 0.9726, respectively.

# References

1. The Chinese Academy for Environmental Planning (2004) The technical key points of surface water's environmental capacity.

2. Li H, Zhe X, Bao B, Han B (2008) Discussion on the degradation of comprehensive attenuation coefficient in river water quality model. Environmental Pollution & Control 6: 1-7.

3. Chen S, Liao W, Xu A, Luo B (1991) Degradation dynamic of COD in sewages in the Xiamen sea area. ACTA OCEANOL SIN 3: 6.

4. Ji M, Sun Z, Wang Z, Tao J (1999) A simulation of the biodegradation process of COD from ocean outfall discharge in Bohai Bay. Oceanologica et Limnologica Sinica 30: 731-736.

5. Li G (2013) The research on pollutant degradation rule in the sewage outlet downstream of three gorges reservior area. Chongqing: Chongqing Jiaotong University.

6. Ventana Systems I Vensim.
