# Supplementary material for: Evaluating the Effects of Land Use Planning for Non-Point Source Pollution Based on a System Dynamics Approach in China
Source: PLoS One. 2015 Aug 12;10(8):e0135572. doi: 10.1371/journal.pone.0135572 (PMC4534394; doi:10.1371/journal.pone.0135572)
Supplement: S5 Text — (DOC) [file pone.0135572.s005.doc]

# S5 Text. Assigning the model

There are five kinds parameters in terms of Land use ratios that account for the total area under different scenarios (LRATA), Runoff coefficient of a given land use type(RCLU), Pollution intensities of different land use types (PILU), Decrement of NPS pollution (DNPSP) and Statistical values (SV) for historical fit test, need to be assigned; different data sources and approaches are used for the assignment, see Table 5 in the manuscript; specific settings were shown in Table A.

**Table A. Assignment of the model**

| **Variable/Parameter** | **Formula/Assignment** | **Unit** | **Parameter sort** |
| --- | --- | --- | --- |
| Amount of runoff in other land for construction | ="Area of other land for construction (AOLC)"*Average amount of annual rainfall*Integrated runoff coefficient of other land for construction | 1000m^3^ |  |
| "Amount of runoff in other land for non-construction" | ="Area of other land for non-construction (AOLNC)" *Average amount of annual rainfall *"Integrated runoff coefficient of other land for non-construction" | 1000 m^3^ |  |
| Amount of runoff in the commercial land | ="Area of commercial land (ACL)"*Average amount of annual rainfall*Integrated runoff coefficient of the commercial land | 1000 m^3^ |  |
| Amount of runoff in the industrial land | ="Area of industrial land (AIL)"*Average amount of annual rainfall*Integrated runoff coefficient of industrial land | 1000 m^3^ |  |
| "Area of agricultural land (AAL)" | ="Total area (TA)"*Ratio of AAL accounts for TA | ha |  |
| "Area of commercial land (ACL)" | ="Total area (TA)"*Ratio of ACL accounts for TA | ha |  |
| "Area of industrial land (AIL)" | ="Total area (TA)"*Ratio of AIL accounts for TA | ha |  |
| "Area of other land for construction (AOLC)" | ="Total area (TA)"*Ratio of AOLC accounts for TA | ha |  |
| "Area of other land for non-construction (AOLNC)" | ="Total area (TA)"*Ratio of AOLNC accounts for TA | ha |  |
| " Total area of land for construction (TALC)" | ="Area of commercial land (ACL)"+"Area of industrial land (AIL)"+"Area of other land for construction (AOLC)" | ha |  |
| " Total Area of land for non-construction (TALNC)" | ="Area of agricultural land (AAL)"+"Area of other land for non-construction (AOLNC)" | ha |  |
| Average amount of annual rainfall | =1.2 | m | SV |
| "Baseline NPSP (BNPSP)" | =44068.2 | ten kg | SV |
| "Decrement of NPSP (DNPSP)" | ="Non-point source pollution (NPSP)"*The integrated rate of DNPSP | ten kg/year |  |
| "Increment of NPSP (INPSP)" | =INPSP in the agricultural land+ INPSP in the commercial land+ INPSP in the industrial land+ INPSP in other land for construction + "INPSP in other land for non-construction " | ten kg/year |  |
| INPSP in other land for construction | =Amount of runoff in other land for construction *NPSP intensity in other land for construction | ten kg |  |
| "INPSP in other land for non-construction " | ="Amount of runoff in other land for non-construction"*"NPSP intensity of other land for non-construction" | ten kg |  |
| INPSP in the agricultural land | ="Area of agricultural land (AAL)"*NPSP intensity in the agricultural land | ten kg |  |
| INPSP in the commercial land | =Amount of runoff in the commercial land*NPSP intensity in the commercial land | ten kg |  |
| INPSP in the industrial land | =Amount of runoff in the industrial land*NPSP intensity in the industrial land | ten kg |  |
| Integrated runoff coefficient of industrial land | =0.7 | Dmnl | RCLU |
| Integrated runoff coefficient of other land for construction | =0.6 | Dmnl | RCLU |
| "Integrated runoff coefficient of other land for non-construction" | =0.15 | Dmnl | RCLU |
| Integrated runoff coefficient of the commercial land | =0.8 | Dmnl | RCLU |
| "Non-point source pollution (NPSP)" | =INTEG ("Increment of NPSP (INPSP)"- "Decrement of NPSP (DNPSP)",44068.2) | ten kg |  |
| NPSP intensity in other land for construction | =71.3 | mg/L | PILU |
| NPSP intensity in the agricultural land | =129.66 | kg/ha | PILU |
| NPSP intensity in the commercial land | =59.78 | mg/L | PILU |
| NPSP intensity in the industrial land | =52.4 | mg/L | PILU |
| "NPSP intensity of other land for non-construction" | =9.62 | mg/L | PILU |
| Ratio of AAL accounts for TA | =  S1: 51.3/(1-0.317*EXP(-0.026*(Time-2010)))/100;  S2: 56.9/(1-0.242*EXP(-0.028*(Time-2010)))/100;  S3: 38.7/(1-0.485*EXP(-0.021*(Time-2010)))/100;  S4: 56.9/(1-0.242*EXP(-0.038*(Time-2010)))/100;  S5: 38.7/(1-0.485*EXP(-0.028*(Time-2010)))/100;  S6: 56.9/(1-0.242*EXP(-0.056*(Time-2010)))/100;  S7: 38.7/(1-0.485*EXP(-0.042*(Time-2010)))/100 | Dmnl | LRATA |
| Ratio of ACL accounts for TA | =  S1: 0.8/(1+1.546*EXP(-0.064*(Time-2010)))/100;  S2: 1.9/(1+5.333*EXP(-0.100*(Time-2010)))/100;  S3: 3.4/(1+10.333*EXP(-0.126*(Time-2010)))/100;  S4: 1.9/(1+5.333*EXP(-0.133*(Time-2010)))/100;  S5: 3.4/(1+10.333*EXP(-0.167*(Time-2010)))/100;  S6: 1.9/(1+5.333*EXP(-0.199*(Time-2010)))/100;  S7: 3.4/(1+10.333*EXP(-0.251*(Time-2010)))/100 | Dmnl | LRATA |
| Ratio of AIL accounts for TA | =  S1: 4.1/(1+1.546*EXP(-0.064*(Time-2010)))/100;  S2: 4.9/(1+2.063*EXP(-0.070*(Time-2010)))/100;  S3: 8.2/(1+4.125*EXP(-0.091*(Time-2010)))/100;  S4: 4.9/(1+2.063*EXP(-0.093*(Time-2010)))/100;  S5: 8.2/(1+4.125*EXP(-0.121*(Time-2010)))/100;  S6: 4.9/(1+2.063*EXP(-0.140*(Time-2010)))/100;  S7: 8.2/(1+4.125*EXP(-0.181*(Time-2010)))/100 | Dmnl | LRATA |
| Ratio of AOCL accounts for TA | =  S1: 34.1/(1+1.546*EXP(-0.064*(Time-2010)))/100;  S2: 23.0/(1+0.729*EXP(-0.050*(Time-2010)))/100;  S3: 40.0/(1+2.008*EXP(-0.069*(Time-2010)))/100;  S4: 23.0/(1+0.729*EXP(-0.067*(Time-2010)))/100;  S5: 40.0/(1+2.008*EXP(-0.093*(Time-2010)))/100;  S6: 23.0/(1+0.729*EXP(-0.100*(Time-2010)))/100;  S7: 40.0/(1+2.008*EXP(-0.139*(Time-2010)))/100 | Dmnl | LRATA |
| Ratio of AONCL accounts for TA | =  S1: 9.6/100;  S2: 13.3/(1+0.385*EXP(-0.043*(Time-2010)))/100;  S3: 9.7/(1+0.010*EXP(-0.035*(Time-2010)))/100;  S4: 13.3/(1+0.385*EXP(-0.058*(Time-2010)))/100;  S5: 9.7/(1+0.010*EXP(-0.047*(Time-2010)))/100;  S6: 13.3/(1+0.385*EXP(-0.087*(Time-2010)))/100;  S7: 9.7/(1+0.010*EXP(-0.070*(Time-2010)))/100 | Dmnl | LRATA |
| "Relative NSP (RNPSP)" | ="Non-point source pollution (NPSP)"/"Baseline NPSP (BNPSP)" | Dmnl |  |
| The integrated rate of DNPSP | =WITH LOOKUP ("Relative NSP (RNPSP)", ([(0,0)-(10,10)],(0.63,0.7331),(1,0.9087),(1.5,0.9543),(2,0.9726),(10,0.9726 ) )) | Dmnl | DNPSP |
| "Total area (TA)" | =2286 | ha | SV |

Note: S1-S7 represents different scenarios (see Table 2 in the manuscript).
